# Visual-induced expectations modulate auditory cortical responses

**DOI:** 10.3389/fnins.2015.00011

**Published:** 2015-02-06

**Authors:** Virginie van Wassenhove, Lukasz Grzeczkowski

**Affiliations:** ^1^CEA, DSV/I^2^BM, NeuroSpin; INSERM, Cognitive Neuroimaging Unit, U992; Université Paris-SudGif-sur-Yvette, France; ^2^Laboratory of Psychophysics, Brain Mind Institute, École Polytechnique Fédérale de LausanneLausanne, Switzerland

**Keywords:** MEG, multisensory, predictive coding, neuronal oscillations, alpha, gamma, eye position, phase-resetting

## Abstract

Active sensing has important consequences on multisensory processing (Schroeder et al., 2010). Here, we asked whether in the absence of saccades, the position of the eyes and the timing of transient color changes of visual stimuli could selectively affect the excitability of auditory cortex by predicting the “where” and the “when” of a sound, respectively. Human participants were recorded with magnetoencephalography (MEG) while maintaining the position of their eyes on the left, right, or center of the screen. Participants counted color changes of the fixation cross while neglecting sounds which could be presented to the left, right, or both ears. First, clear alpha power increases were observed in auditory cortices, consistent with participants' attention directed to visual inputs. Second, color changes elicited robust modulations of auditory cortex responses (“when” prediction) seen as ramping activity, early alpha phase-locked responses, and enhanced high-gamma band responses in the contralateral side of sound presentation. Third, no modulations of auditory evoked or oscillatory activity were found to be specific to eye position. Altogether, our results suggest that visual transience can automatically elicit a prediction of “when” a sound will occur by changing the excitability of auditory cortices irrespective of the attended modality, eye position or spatial congruency of auditory and visual events. To the contrary, auditory cortical responses were not significantly affected by eye position suggesting that “where” predictions may require active sensing or saccadic reset to modulate auditory cortex responses, notably in the absence of spatial orientation to sounds.

## Introduction

In a vast majority of psychological and neuroimaging paradigms, participants' eyes position is maintained on a fixation cross located straight in front of them. However, in natural settings, active sensing (Schroeder et al., 2010) yields organisms to reorient their gaze or themselves (Maier and Groh, 2009) so as to privilege the sampling of relevant multisensory information in space and in time. Reorienting tends to be automatic: in dichotic listening tasks, naïve participants naturally make eye movements toward the sound source (Gopher and Kahneman, 1971) as information sampling in one sensory modality can affect the processing in another sensory modality, notably during complex ecological scene analysis (Zion Golumbic et al., 2012). The position of the eyes is known to affect auditory spatial localization (Lewald and Ehrenstein, 1996; Maddox et al., 2014) and more generally audiovisual integration (Hartnagel et al., 2007). Still, if eye positions tend to correlate with the focus of spatial attention (Yarbus, 1967), they are also largely dissociable from covert spatial attention (Posner, 1980; Desimone and Duncan, 1995).

In monkey neurophysiology, neural responses at different stages of auditory processing (Jay and Sparks, 1984; Werner-Reiss et al., 2003; Mullette-Gillman et al., 2005; Bulkin and Groh, 2006), including primary auditory cortex (Werner-Reiss et al., 2003; Fu et al., 2004), are known to be modulated by eye positions. Whether the nature of these modulations is purely feed-forward (Werner-Reiss et al., 2003) or driven by attention and feedback projections (Fu et al., 2001, 2003) remains unknown; it is also unclear whether eye positions *per se* or shifts in attention may be at the origin of modulatory effects in auditory neural responses. By far, only one fMRI study in humans has suggested a right-hemispheric dominance modulated by the spatial incongruence of eye positions and sound source (Petit et al., 2007) although several studies have highlighted the importance of supramodal attention under such conditions (e.g., Banerjee et al., 2011).

**Figure 1:**
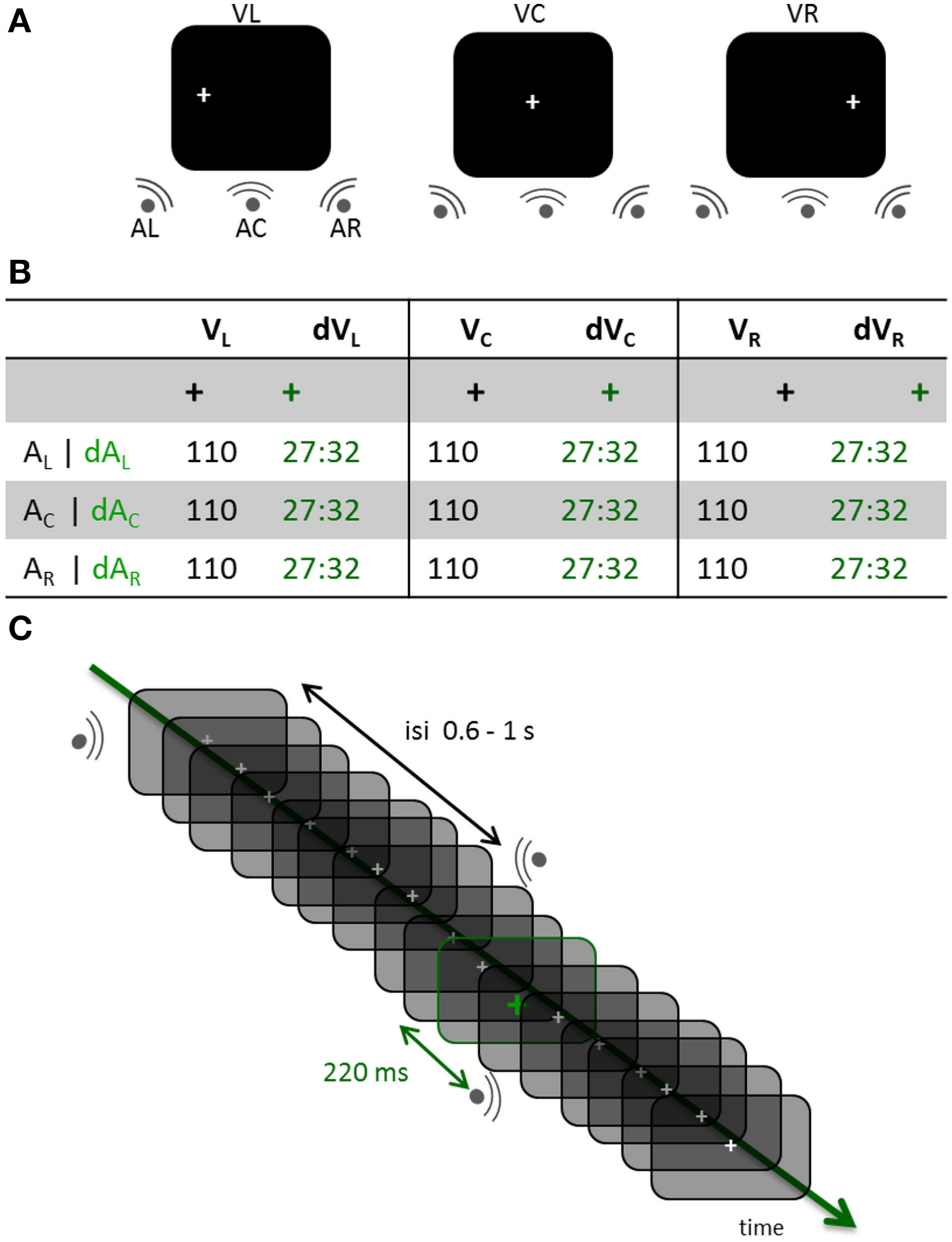
**Experimental design. (A)** Three distinct experimental blocks were run across participants. In each block, participants had to maintain the position of their eyes on a fixation cross located to the left, the center or the right side of the screen (V_L_, V_C_, and V_R_, respectively). Within each block, sounds were randomly displayed on the left, center or right side of the participant (A_L_, A_C_, and A_R_, respectively). **(B)** In all experimental blocks, a visual oddball design was used consisting of the gray fixation cross turning green in about 30% of the trials. In the standard (STD) conditions, the fixation cross did not change color prior to a sound being played; trials in which the fixation cross turned green were deviant trials (DEV). All possible combinations of gaze directions and sound locations were tested in the STD and DEV conditions. Participants counted the number of green crosses within each block (jittered randomly across blocks between 81 and 96). **(C)** DEV and STD trials of all three possible sound positions were intermixed within a block. The inter-stimulus-interval (ISI) was pseudo-randomly chosen between 0.6 and 1 s. When the fixation cross turned green (DEV trial), the subsequent sound systematically occurred 220 ms later irrespective of its location.

Here, we used a visual oddball paradigm with magnetoencephalography (MEG) and asked whether maintaining the position of the eyes fixed (i.e., not preceded or followed by a saccade) would be sufficient to automatically affect auditory cortical responses while participants were engaged in a visual task. The task consisted in keeping track of the number of colour changes of the fixation cross in a given block while maintaining the position of the eyes to the right, the left or the centred fixation point (V_R_, V_L_ and V_C_, respectively). Meanwhile, task irrelevant noise bursts were played at variable locations (left, right or center; A_L_, A_R_ and A_C_, respectively). Trials in which the fixation cross did not change color were standard trials (STD); trials in which the fixation cross turned green 220 ms before a sound were deviant trials (DEV, ~30%). Note that the deviance affected the color of the fixation cross irrespective of the spatialized sounds which were equally probable in each eye positions block, and in both STD and DEV conditions. Hence, nine combinations of eye positions and sound locations were tested in both STD and DEV trials (Figure 1). This design allowed contrasting the effect of visual “when” predictions (namely, a color change systematically predicting the presentation of a sound 220 ms later irrespective of its location) and “where” predictions (would the position of the eyes automatically orient auditory attention to that location in space) on early auditory cortex responses. We asked whether eye position selectively affected early auditory responses by separating STD and DEV trials according to the spatial congruency between sound location and eye position.

Auditory evoked and oscillatory activities were analyzed and showed no clear evidence of early auditory response modulations by eye position; to the contrary, systematic modulations of the auditory responses were found according to the high temporal predictability of visual color changes. These results suggest that in the absence of overt spatial attention to audition, steady eye positioning does not significantly modulate early or pre-stimulus auditory response as captured with MEG whereas transient color changes do.

## Material and methods

### Participants

Fourteen healthy participants (mean age of 23 years old) took part in the study. None had any known neurological or psychiatric disorder and all had normal hearing and normal or corrected-to-normal vision. Three participants were taken out of the study due to low signal-to-noise and contamination by eye movement artifacts. Written informed consents were obtained from each participant in accordance with the Declaration of Helsinki (2008) and the Ethics Committee on Human Research at the Commissariat à l'Energie Atomique et aux Energies Alternatives (CEA, DSV/I^2^BM, NeuroSpin, Gif-sur-Yvette, France). All participants were compensated for their participation.

### Stimuli

Auditory stimuli consisted of 40 ms white noise bursts (5 ms on and off ramping) presented binaurally (central condition, A_c_) or monaurally (left or right side, A_L_ or A_R_respectively). Inter-stimulus intervals (ISIs) were pseudo-randomly chosen from a uniform distribution ranging from 660 to 1020 ms on a trial-per-trial basis. All sounds were delivered at a comfortable hearing level through Etymotic earplugs (~65 dB). A white fixation cross was continuously displayed on the left (V_L_), center (V_C_) or right (V_R_) side of the screen; the eccentricity for V_L_ and V_R_ was 11° of visual angle. In a given run, the visual fixation cross remained at the same location while sounds were randomly presented in each of the three possible locations (Figure 1A). About 30% of the time (jittered randomly across blocks, between 81 and 96 events per block), the white visual cross turned green for 48 ms (Figure 1B). Participants were asked to keep track of the total number of green crosses within a block and to report their count at the end of the block. A visual color change was systematically followed by the presentation of a sound 220 ms later (Figure 1C). All participants performed above 90% chance on the task. Stimuli were presented using Psychtoolbox (Brainard, 1997).

### Procedure

After written consent, participants were asked to change in pajamas to avoid any magnetic artifact in the MEG. The ECG (electrocardiogram, 3 electrodes), EOG (electrooculogram, four electrodes) and HPI coils (Head Position Coils, four coils) were placed at their respective locations by trained nurses and the experimenters. The anatomical landmarks (nasion and preauricular points), the position of the HPI coils and participants' head shape were digitized (Polhemus Isotrak system). Participants were brought into the MEG magnetic-shielded room, comfortably seated and explained the task. They were told to keep their eyes open during the presentation of the stimuli and to maintain the position of their head as still as possible. This was facilitated by the use of an amagnetic chin rest fixed onto the MEG dewar. Participants were told to blink during the rest intervals if and when needed. The eye tracker (Eyelink, SR Research, Canada) and sound level were calibrated prior to the MEG recordings. Participants were encouraged to ask any question prior to the experiment. Each run lasted no more than 10 min for a total of 45 min (including breaks).

### MEG recordings

Brain activity was recorded with a 306-channel Neuromag system (Elekta-Neuromag Oy; Helsinki, Finland) in a magnetically shielded room (Euroshield Oy, Eura, Finland) at NeuroSpin (CEA, DSV/I^2^BM, France). The MEG device includes two orthogonal planar gradiometers and one magnetometer per sensor unit for a total of 204 planar gradiometers and 102 magnetometers. Prior to each experimental run, the position of the participant's head in the MEG dewar was measured by feeding the HPI coils with distinctive currents prior to actual brain measurements. The ECG and EOG (horizontal and vertical) were simultaneously recorded for artifact corrections and trial rejections (see pre-processing). Data were acquired with a sampling frequency of 1 kHz, low-pass filtered at 330 Hz and high-pass-filtered at 0.1 Hz.

### Eye tracker recordings

An MEG-compatible eye tracker simultaneously monitored participants' eye position (Eyelink 1000; SR Research Ltd., Mississauga, Ontario, Canada). The eye-tracker was used monocularly (right eye) to insure that participants properly maintained eye positions steadily on the cross as instructed. The eye tracker was calibrated before each run.

### Anatomical MRI and MEG-MRI coregistration

Anatomical T1-weighted MRIs were obtained for each participant with a 3T MRI scanner (Siemens) with 1 × 1 × 1.1 mm resolution. Digitized anatomical landmarks, HPI and head shape information were used for proper realignment of MEG data with each individual' MRI. The coregistration was performed using both mrilab and mne_analyze.

### Forward model

MRI segmentation was performed using FreeSurfer (v5.1.0, RRID: nif-0000-00304). Surfaces of the Boundary Elements Model (BEM) were constructed using MNE (v2.7.3, MNE - Minimum Norm Current Estimates, RRID: nlx_151346) and the mne_watershed_bem command. Surfaces were manually checked using Freesurfer (v5.1.0, RRID: nif-0000-00304). Source models were done with loose orientation (mne_setup_source_space –ico 6) and mne_setup_forward_model using 5120 vertices per hemisphere and BEM layer (one layer).

### MEG data preprocessing

Data were pre-processed in two steps. First, magnetic interferences originating outside of the MEG helmet were suppressed by using Signal Space Separation (Taulu and Simola, 2006) provided by MaxFilter (Elekta-Neuromag Oy; Helsinki, Finland). The median head position of each participant over the three experimental runs was used as reference for the other two runs. In the majority of cases, the second run was the reference run. Second, PCA was performed to remove components accounting for ECG and EOG variance using Graph (Elekta-Neuromag Oy; Helsinki, Finland). The average cardiac and blink artifacts were computed on the basis of ECG and EOG recordings. Components were manually checked for each sensor type (gradiometers and magnetometers) and saved as separate matrices (for detailed procedure, see: http://www.unicog.org/pm/pmwiki.php/MEG/RemovingArtifactsWithPCAAndGRAPH).

### Source reconstruction and data processing

Data were processed using in-house MNE-python pipelines elaborated on existing procedures (http://mne-tools.github.com/mne-python-intro/, RRID: SciRes_000118). Continuous data were segmented into 1 s epochs centered on auditory stimulus onsets from −400 to 600 ms post-auditory stimulus onset. Baseline correction was applied from −400 to −250 ms before the auditory onset (i.e., −400 to −30 ms with respect to the visual onset for DEV stimuli). Epochs were averaged per condition of interest and source reconstructed on the whole cortex (dSPM) to provide the temporal course of source estimates.

For time-frequency analysis, a Morlet wavelet transform was used on single trial source estimates from 2 to 120 Hz. Data were decimated three times (i.e., new sampling frequency of 333 Hz) and computed as a percentage change from baseline (Kiebel et al., 2005). The width of the wavelet was scaled with frequency (from 4 to 120 Hz in 2 Hz steps) so that 3 cycles were used per frequency step (number of cycles = [4:120]/3).

In both evoked and time-frequency analyses, a third of the STD trials was used in the comparison of STD vs. DEV in order to equate the number of epochs in the noise normalization of dSPM for each condition of interest. All epochs were preserved when comparing STD conditions among themselves. The noise covariance matrix was built using all baselines extracted from all conditions. Auditory cortex labels were manually and individually defined on the morphed freesurfer averaged brain on a per individual basis by using the grand average data of centered gaze (V_C_) with centered sound (A_C_). FreeSurfer parcellation was otherwise used as indicated in text (e.g., Transverse Temporal Gyrus or TTG label).

### Statistical analysis

Statistical analyses performed in sensor space used FieldTrip routines and analyses in source estimates used MNE-python. Both analyses used cluster-level permutation tests temporally or spatiotemporally (Maris and Oostenveld, 2007; Oostenveld et al., 2011). The number of permutations applied was 1024. For source estimates, an epsilon value of 0. 1 was added in order to correct for spurious and transient variance shifts leading to clusters splitting. Detailed examples of the code can be found here: http://martinos.org/mne/auto_examples/stats/plot_cluster_stats_spatio_temporal.html#example-stats-plot-cluster-stats-spatio-temporal-py.

All significant results are reported for t values of 3.13 and corrected p values <0.01 unless otherwise specified (for instance, lower *t* = 2.2 for corrected *p* values <0.05 were also tested). Statistical analyses of time-frequency contrasts were realized using cluster permutations (500) over the full time-frequency spectrum. Reported effects are based on frequency regions defined as: theta (4–7 Hz), alpha (8–12 Hz), low beta (beta1: 14–18 Hz), high beta (beta2: 20–30 Hz), low gamma (gamma1: 32–62 Hz) and high gamma (gamma2: 64–120 Hz).

## Results

### Auditory cortex responses to lateralized sounds

**Figure 2:**
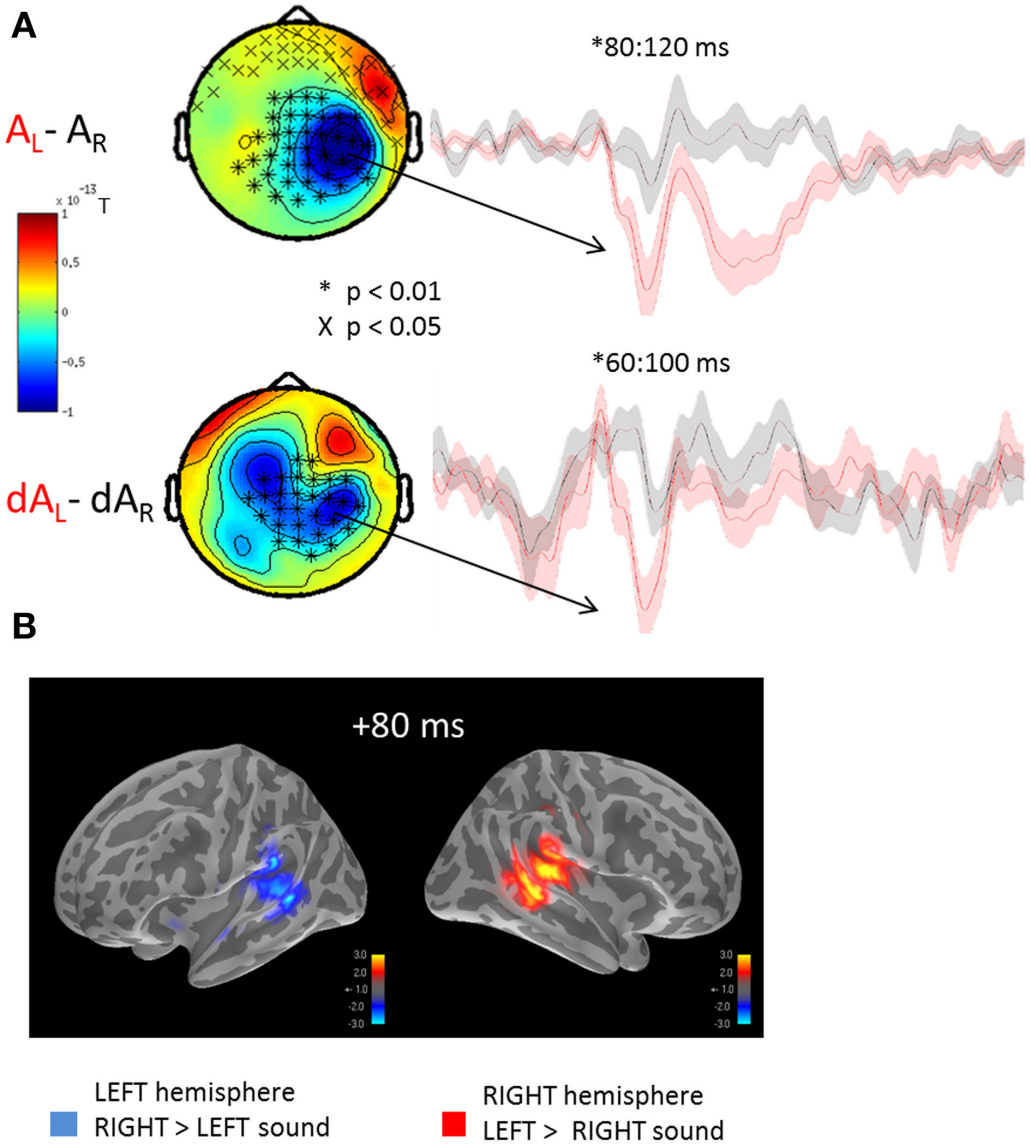
**Mean auditory fields and source estimates evoked by the presentation of lateralized sounds irrespective of eye position**. **(A)** Auditory responses evoked by the presentation of a left (red trace) or right (black trace) sound irrespective of eye positions were contrasted and submitted to cluster analysis for both STD (top row, AL-AR) and DEV trials (bottom row, dAL-dAR). The topographies (left panels) highlight the significant sensor clusters found in the contrasts, namely here: a left sound evoked a larger response in the right hemispheric sensors. Two observations can be made: whereas a left sound significantly increased the amplitude of the auditory evoked response in the right hemisphere, a right sound did not significantly increase the response in the left hemisphere. This assymetry was captured in source space as well cf. **(B)**. Although a similar topography can be seen in STD and DEV trials, additional sources appear to contribute the differential responses in DEV trials. The later difference (~200 ms) observed in the STD trials was not observed in the DEV trials. Additionally, the significant clusters in DEV trials occured slightly earlier than in the STD trials. **(B)** Grand average source estimates (dSPM) contrasting the auditory evoked responses obtained to the presentation of a left and right STD sound (AL-AR) irrespective of eye position (i.e., source-reconstructed contrast from Figure 2A, first row). Consistent with the clusters of evoked responses observed in sensor space, source estimates at a latency of ~80 ms were significantly larger in the right auditory cortices when the sound was presented to the left; conversely, an increased amplitude of the m100 response was observed in the left auditory cortices to the presentation of a right lateralized sound although not significant.

The auditory evoked fields to the presentation of left or right sounds irrespective of the yee positions are illustrated in Figure 2. Using cluster analysis in sensor space (Figure 2A) and whole-brain spatiotemporal clustering in source space (Figure 2B), significant hemispheric differences were found in early auditory evoked responses, namely: a sound presented to the left ear (A_L_) evoked a significantly larger right-lateralized response than a sound presented to the right ear (A_R_) (Figure 2B: significant source estimates differences from 80 to 100 ms, corrected *p* < 0.01). Although sounds presented to the right ear produced larger left-lateralized responses than did sounds presented to left ear, this contrast did not reach significance after correction for multiple comparisons. This pattern of activation is consistent with previous reports in which the auditory m100 component has been shown to be up to 30% larger over the contralateral auditory cortex for monaural sounds (Pantev et al., 1986; Mäkelä et al., 1993). The lack of a significant difference in the left-lateralized response to sounds presented to the right ear may result from the higher ratio of neurons tuned to contra- vs. ipsi-lateral sounds in the right vs. the left hemisphere (Salminen et al., 2010).

### Visual transience modulates baseline activity in auditory cortices

**Figure 3:**
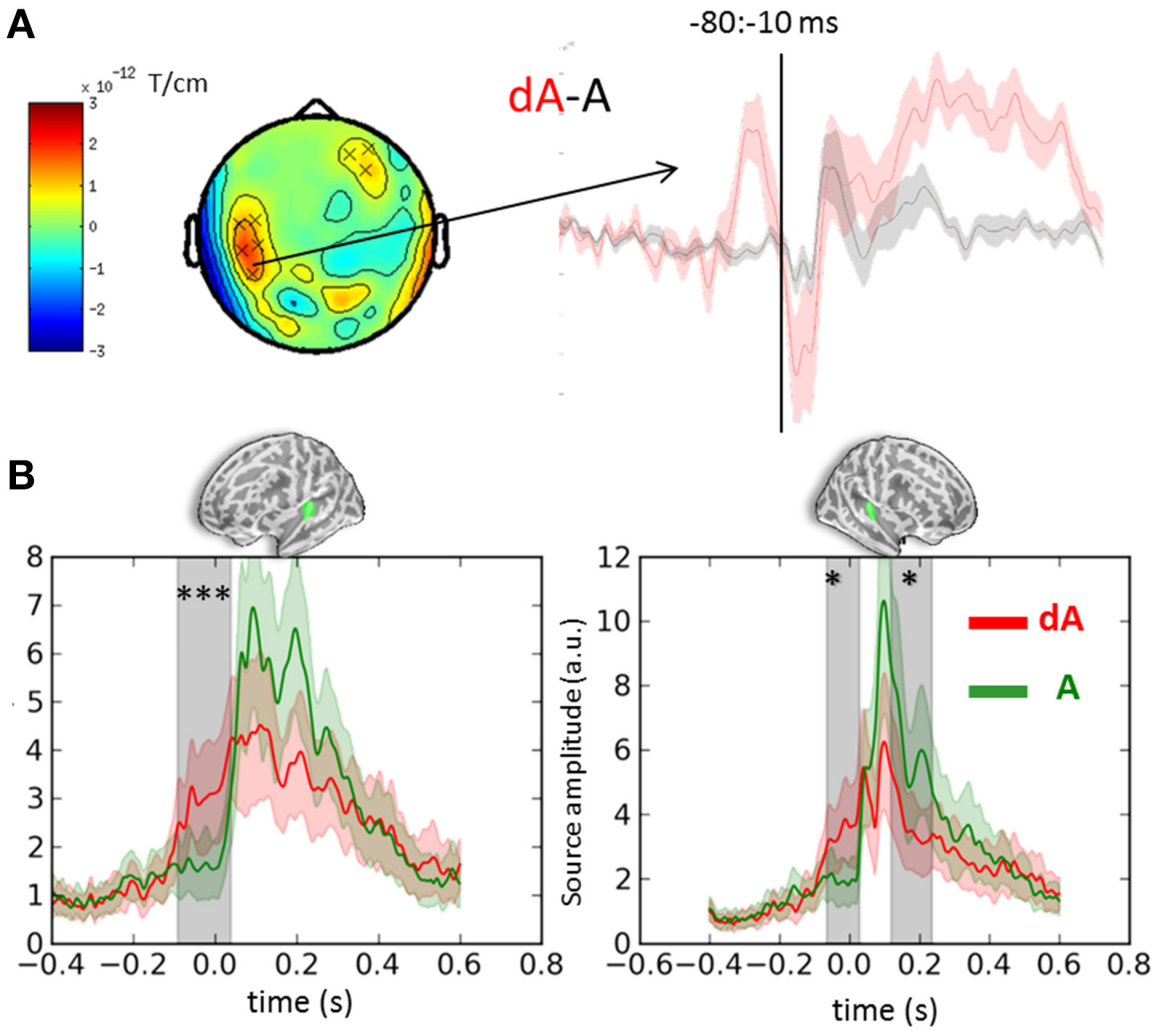
**Event-related-fields and souce estimates for DEV and STD sounds. (A)** Evoked response contrasts of DEV (dA) minus STD **(A)** trials irrespective of eye position (for illustration, gradiometer dx is shown). The dA-A contrast revealed a significant cluster ranging from −80 to −10 ms prior to sound onset. The time-course of the significant sensors (x) are plotted on the right showing a clear evoked response preceding the onset of a sound in the DEV response (dA: red) as compared to the STD trials. **(B)** Mean auditory source estimates in left and right auditory cortices (left and right panels, respectively) of DEV (dA: red) and STD (A: green) trials. Consistent with sensor data in panel **(A)**, temporal cluster analysis in source space revealed significant temporal clusters (gray shaded areas) in both auditory labels (green label above graphs). The earliest significance was observed 100 ms prior to auditory onset in both hemispheres, illustrating a modulation of pre-stimulus activity in auditory cortices following a visual transient color change. Color shaded areas are two s.e.m. ^***^corrected *p* < 10^−3^; ^*^corrected *p* < 0.05.

Cluster analyses were performed in both sensor and source space to contrast the auditory evoked responses obtained in DEV and STD trials irrespective of auditory location and eye positions (dA vs. A, respectively). Both analyses were consistent and revealed a significant bilateral ramping of activity in auditory cortices starting as early as 100 ms prior to the onset of the sound (Figure 3). The auditory source estimates in response to the presentation of DEV trials (Figure 3B, red traces) were significantly smaller in amplitude compared to those obtained in the STD trials (Figure 3B, green traces). As a reminder, DEV trials were associated with a color change in the visual fixation cross and all auditory locations had equal probability of occurrence within each block. As such, no auditory mismatch was expected on the basis of the side of presentation of a sound. Significant clusters (*p* < 0.01, not shown) in the ventral visual stream were also observed, consistent with the processing of a green visual cross in DEV.

Importantly, no significant differences were observed in sensor or source space when contrasting the auditory evoked responses as a function of congruency in auditory location and eye position whether in STD or DEV trials (i.e., (d)A_L_V_L_ vs. (d)A_L_V_R_ or (d)A_R_V_R_ vs. (d)A_R_V_L_) even at lower thresholds. No significant effect of eye position on ongoing or early auditory evoked responses could be found. Time-frequency analyses were performed to address whether oscillatory activity in auditory cortices would carry any relevant information with regards to eye position that evoked response alone would not capture.

### Oscillatory activity irrespective of eye position

**Figure 4:**
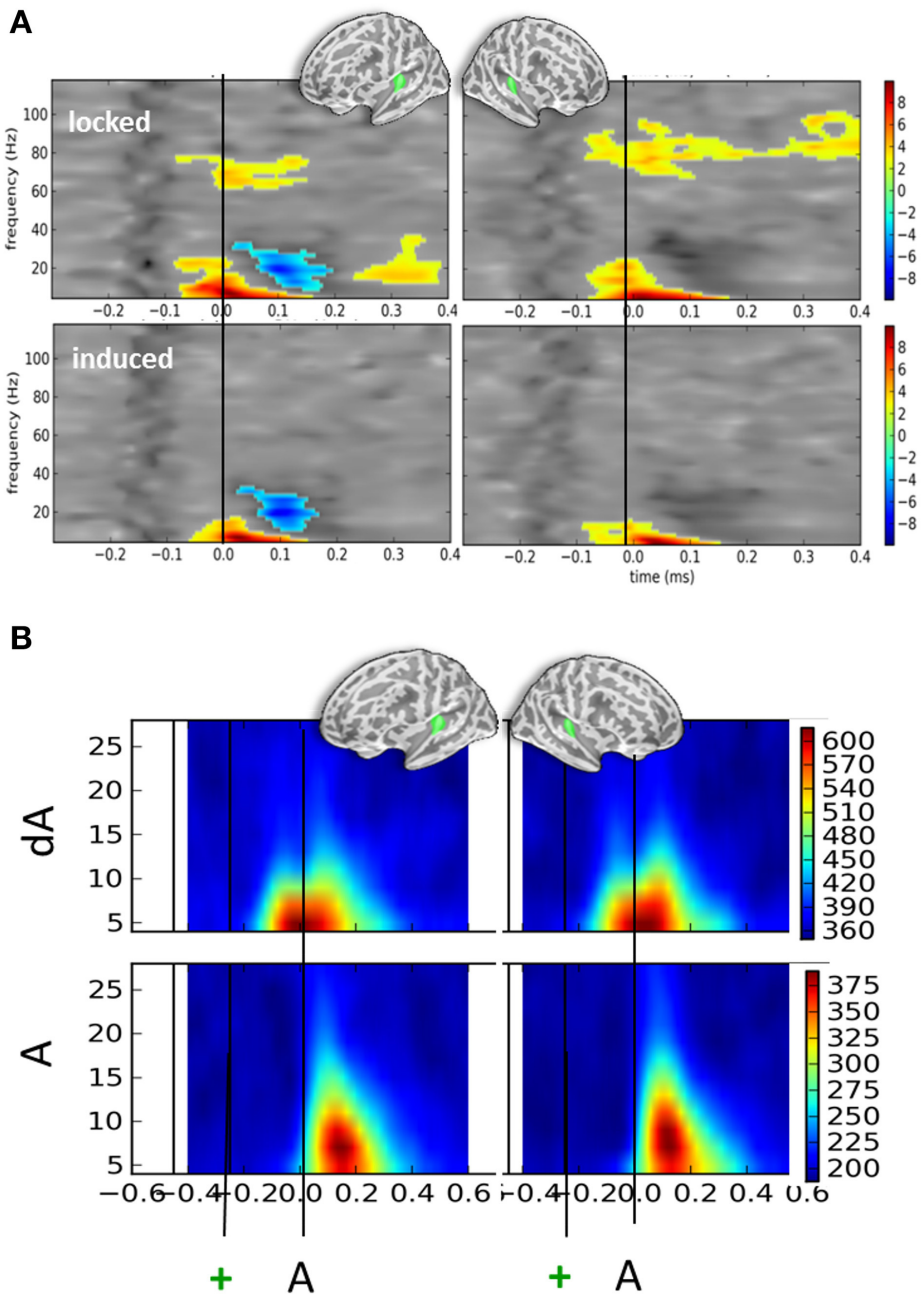
**Time-frequency contrasts of all DEV vs. STD trials irrespective of auditory location or eye position. (A)** T-maps of significant time-frequency clusters contrasting all DEV trials (dA) minus all STD trials (A) in left and right auditory cortices (left and right panels, respectively). The top panels illustrate the contrasts using typical time-frequency analysis on single trials thereby including a mix of phase-locked and induced activities (“locked”). The bottom panels illustrate the same dA-A contrast: in this analysis, the average evoked response was removed from the single trials prior to classic time-frequency analysis (see Methods). This amounts to drastically removing phase-locked activity (“induced”). In both cases, significant temporal clusters starting prior to sound onset (black line) were seen. In DEV trials, a significant increase of alpha and beta power was observed as compared to STD trials. An additional significant decrease of beta power was observed in the left auditory cortices. In the “locked” panels, a significant increase of high gamma power (~80 Hz) was seen bilaterally suggesting that sounds preceded by a transient color change (DEV, dA trials) evoked more high-gamma power than those that were not (STD, A trials). The bilateral increase of alpha power observed in auditory cortices is consistent with attention oriented toward counting visual events in this task. **(B)** Phase-locking values (PLV) observed in DEV (dA, top) and STD (A, bottom) trials. Note that alpha PLV are much higher and occur earlier in dA as compared to A. Actual scaling for PLV should be divived by 1000 (i.e., 600 on the scale corresponds to a PLV of 0.6).

Irrespective of eye position and sound location, significant oscillatory activities were found when contrasting auditory responses to the presentation of DEV (dA) and STD (A) trials (Figure 4A). T-maps of non-parametric cluster analyses results performed on the full time-frequency spectra are provided in Figure 4A for the left and right auditory cortices (top left and right panels, respectively). First, a significant increase of alpha and beta power was observed starting earlier than or around the auditory onset. Temporal cluster analyses contrasting the alpha power in dA and A trials revealed significant effects (corrected *p* < 0.001) ranging from −122 to 33 ms and −206 to 60 ms post-sound onset in the left and right hemispheres, respectively. A similar analysis performed on beta power revealed a significant cluster (corrected *p* < 0.001) from −99 to +37 ms and −151 to −17 ms post-auditory onset in the left and right hemispheres, respectively. A significant beta power decrease was found in the left auditory cortices from 103 to 215 ms post-auditory onset. Additionally, a significant sustained higher high-gamma power (~70 to 80 Hz) was also observed in dA trials as compared to STD trials starting ~100 ms before sound onset in both hemispheres.

Figure 4B provides the associated phase-locking values (PLV or equivalently here, Inter-Trial Coherence) in auditory cortices for DEV (top) and STD (bottom): as can readily be seen, PLV were twice as strong in DEV as compared to those observed in STD trials. Importantly, a shift in the latency of maximal PLV can readily be noted in dA as compared to A. This observation converges with the earlier significant effects observed in the auditory evoked responses (Figure 2A) and suggests that one possible effect of visual color change on auditory response is the phase-resetting of the ongoing alpha component in auditory cortices.

In the bottom panels of Figure 4A, we report the time-frequency contrasts (dA—A) performed on single-trials, this time, after subtraction of the evoked components. This procedure was used to try and dissociate the *evoked* from the *induced* oscillatory activity. This method eliminated the high gamma band response observed in Figure 4A, upper panels, suggesting that the high gamma oscillatory component is to a great extent stimulus-locked. As the high-gamma component observed in Figure 4A (top panels) was found to be significant *prior to* the sound onset, this also suggests that a temporally-informative visual color change contributes to the modulation of gamma-locked auditory response.

### Oscillatory activity as a function of sound location

**Figure 5:**
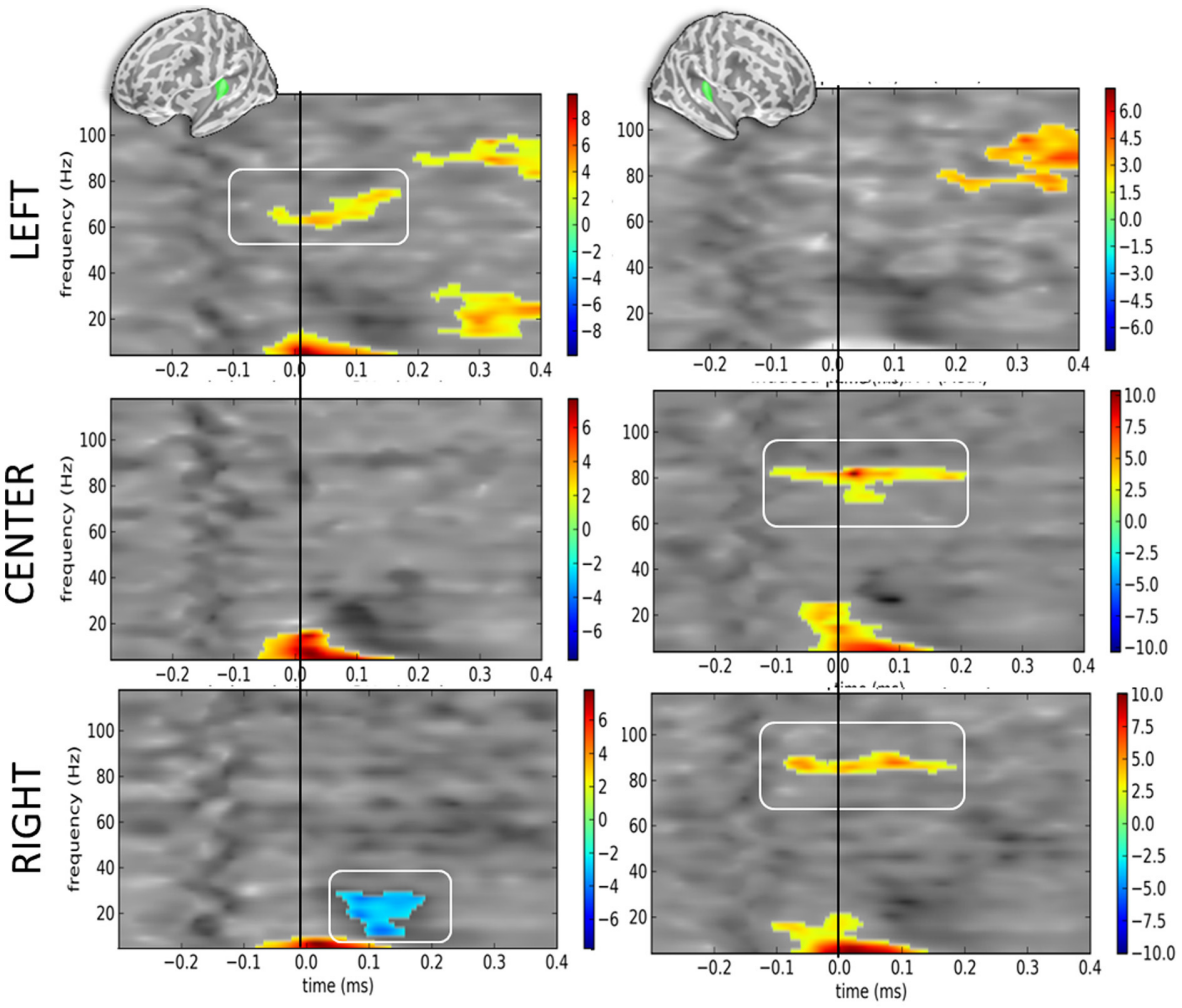
**Time-frequency t-maps of DEV vs. STD contrasts as a function of sound location**. Each graph reports the t-maps of the DEV minus STD time-frequency contrasts separately for the left and right hemispheres (left and right panels, respectively) and as a function of sound location. **Top panels**: ≪ LEFT ≫ are sounds presented to the left ear: t-maps report the contrast dAL minus AL irrespective of eye positions. During DEV trials, sounds presented to the left ear (dAL) elicited a significant increase of high-gamma power as compared to the same sounds during STD trials (AL). This was only observed in the contralateral auditory cortices, here the left hemisphere. **Middle panels**: ≪ CENTER ≫ are sounds presented to both ears: t-maps report the contrast dAC minus AC irrespective of eye positions. A significant increase of high-gamma power was observed in the right auditory cortices in DEV trials (dAC) as compared to STD trials (AC). **Bottom panels**: ≪ RIGHT ≫ are sounds presented to the right ear: t-maps report the contrast dAR minus AR. DEV sounds presented to the right ear (dAR) elicited a significant increase of high-gamma band power in the right auditory cortices as compared to STD trials. Additionally, a significant beta power decrease was observed in the left auditory cortices.

When contrasting DEV vs. STD as a function of sound location but irrespective of eye position (Figure 5), the corrected t-maps of time-frequency contrasts replicated the significant bilateral increase of the alpha component irrespective of sound location—excepted for the right hemisphere when sounds were presented to the left ear (Figure 5, top right panel).

Additionally, the high-gamma frequency component in DEV was significantly bigger in the hemisphere contralateral to the presentation of the sound. For instance (Figure 5, top panels), when a DEV sound was delivered to the left ear irrespective of participants' eye position (dA_L_), a significant power increase in the high-gamma band (~80 Hz) was observed in left auditory cortices as compared to a sound delivered to the left ear without being preceded by a visual color change (A_L_). For centered sounds (dA_C_—A_C_; middle panels), a significant power increase of high-gamma component was seen in the right but not in the left auditory cortices. For sounds presented to the right ear (dA_R_—A_R_; bottom panels), a significant increase of high gamma power was observed in the right auditory cortices but not in the left auditory cortex. In this contrast, a significant decrease in beta power was also seen in the left hemisphere.

Considering that contrasts were performed irrespective of eye position, the lateralized response of high-gamma oscillatory component is likely and mainly driven by auditory location, not by eye position. In DEV-STD contrasts however, the precedence of a transient color change suggests that the significant increase of high-gamma power in DEV trials are nevertheless modulated by a transient change in visual inputs.

### No specific effect of eye position on auditory cortex response

Comparisons specifically addressing the effect of eye position on auditory cortex response namely: STD (V_L_-V_R_) or DEV (dV_L_-dV_R_) yielded no significant results in evoked or oscillatory response. Similarly, neither evoked nor time-frequency analysis showed any systematic interaction between eye position and sound location, namely: hearing a sound on the left and looking on the right vs. looking on the left (dA_L_V_R_–dA_L_V_L_) or hearing a sound on the right and looking on the left vs. looking on the right (dA_R_V_L_–dA_R_V_R_) showed no significant cluster. Altogether, no reliable or systematic modulation of eye position on early auditory cortex response was found in this experiment.

## Discussion

In this study, we asked whether maintaining the eye positions in a particular direction would affect auditory cortex response to different sound locations (“where” prediction) and whether a temporally predictive color change would affect auditory response irrespective of sound location (“when” prediction”). We found that transient visual color changes systematically affected auditory cortex responses bilaterally: an increased ramping activity preceding sound onset and a bilateral decrease of auditory evoked responses to the presentation of the sound were observed. Consistent with the ramping activity preceding sound onset, an early increase of phase-locking value was found during deviant trials presentation. Second, time-frequency analysis revealed a systematic increase of alpha and high-gamma band power around auditory onset in DEV trials as compared to STD trials. Third, significant high gamma-band responses tended to be contralateral to the sound location in DEV trials, suggesting a possible gain modulation of the lateralized auditory response by transient visual color changes. These effects were independent of eye position and no systematic and specific modulations of auditory evoked or oscillatory responses as a function of participant's eye positions were observed in this experiment.

### Right-hemispheric lateralization of spatialized sounds

Spatialized sounds are known to elicit asymmetric responses in auditory cortices whether sounds are presented monaurally (Reite et al., 1981; Mäkelä et al., 1993) or binaurally (McEvoy et al., 1993; Sams et al., 1993; Loveless et al., 1994); but see (Woldorff et al., 1999). The m100 component has been shown to be up to 30% larger over the contralateral auditory cortex for monaural sounds (Pantev et al., 1986; Mäkelä et al., 1993), and this difference notably affected the right hemisphere. At the origin of this difference, one working hypothesis is that the ratio of neurons tuned to sound sources in the contralateral vs. ipsilateral hemifield is higher in the right than in the left hemisphere (Salminen et al., 2010). As such, right hemispheric responses to left lateralized sounds have been shown to be larger than the left hemispheric responses to right auditory sources. Similarly here, significant right hemispheric differences could be found for monaural sounds presented to the left but the trend for left hemispheric increase did not reach significance for monaural sounds presented to the right ear.

### Visual “where” information to auditory cortex

In many species, non-auditory inputs have been found to modulate the response properties of auditory neurons throughout the auditory pathway (Cahill et al., 1996; Schroeder et al., 2001; Wallace et al., 2004; Cappe and Barone, 2005; Ghazanfar et al., 2005; Budinger et al., 2006; Bizley et al., 2007; Lakatos et al., 2007; Bizley and King, 2008). In auditory association cortices, visual modulations are mediated by feedback and lateral projections as defined by laminar profiling and anatomical connectivity (Rockland and Pandya, 1979; Felleman and van Essen, 1991; Rockland and Ojima, 2003; van Wassenhove and Schroeder, 2012). Non-specific feed-forward projections via koniocellular neurons have also been mentioned to potentially contribute to these modulations (Fu et al., 2003; Schroeder et al., 2003).

One goal of the study was to assess whether eye positions in the absence of saccades and while paying attention to vision would automatically modulate auditory cortex responses to spatialized sounds. In other words, can eye position automatically direct attention to a congruent sound source (e.g., looking on the right would enhance attention to the sound that effectively arrives on the right side) as ventriloquist effects and recent perceptual effects would suggest (Bonath et al., 2007). In this study, no clear modulation of the auditory evoked responses (whether in sensor or source space) were found based on eye position alone: the response pattern to spatialized sounds was similar in both auditory cortices irrespective of eye position, congruency between eye position and sound location (STD trials) or congruency between transient visual events location and sound location (DEV trials). These results suggest that, at least in the absence of overt spatial attention directed to audition and in the absence of transient reset of eye position (blink, saccade), the eye positions do not selectively modulate early auditory cortex responses.

It is noteworthy that in a previous EEG study (Teder-Sälejärvi and Hillyard, 1998), increased amplitudes of the auditory evoked responses were found for attended sound sources. Here, contrary to this EEG study, auditory stimuli were task-irrelevant and unattended. One possibility is thus that when participants are engaged in an auditory spatial judgment task (e.g., Bonath et al., 2007), eye positions readily bias activity in auditory cortices. These results support the notion that eye position is not sufficient to direct (supramodal) attention and is dissociable from covert attention. These results thus converge with prior studies highlighting the importance of top-down spatial attention in the modulation of auditory evoked responses (Banerjee et al., 2011).

### Cautionary notes on the lack of eye positions effects in auditory response and limitations of the study

Several factors may have limited the possibility to observe a clear influence of eye positions on auditory cortex responses in this task and with this functional neuroimaging technique. First, the estimates of the proportion of auditory neurons sensitive to eye positions are variable throughout the auditory pathway. Of particular relevance here, single cell recordings in monkeys reported that the excitability of roughly 12% of neurons in auditory cortex was modulated by eye positions (Werner-Reiss et al., 2003; Fu et al., 2004). This small percentage together with the location, concentration and orientation of the contributing neural sources in human auditory cortex may have prevented seeing a clear modulation in the MEG signals. As such, future work should address these issues by optimizing the experimental design and by increasing the number of relevant contrasting trials.

Second, the position of the eyes was maintained in a given direction throughout an experimental block so that no saccade or shift of position occurred across trials. This design contrasts with prior studies in which a shift in the position of the eyes could occur on every trial (e.g., Maier and Groh, 2010) suggesting that transient shifts in the position of the eyes may be an important factor for the observation of modulatory effects in auditory cortex responses.

Third, the current experimental design did not make use of head-related transfer functions for sound displays. Classic multisensory integration rules predict that optimal audiovisual integration occurs when preserving a spatiotemporal mapping across sensory modalities (Stein and Meredith, 1993). However, the existence of windows of integration may relax the need for precise spatiotemporal mapping in cortex; it is nevertheless plausible that more realistic rendering of the stimuli may allow for clearer and stronger responses across sensory modalities.

Fourth, it could be argued that since direct connectivity between auditory and visual cortices entails peripheral vision as shown by neuroanatomical studies (Falchier et al., 2002; Rockland and Ojima, 2003), foveal fixation may have prevented modulatory effects. We think that this is unlikely because this should hold for neurophysiological studies which have reported effective modulations using foveal fixation.

### Alpha increase in auditory cortices as active suppression of incoming information

Ongoing activity preceding the presentation of a stimulus has been reported in several studies and are considered to be predictive of the behavioral outcome in the context of audiovisual integration (Keil et al., 2012). Whether fluctuations in the pre-stimulus baseline reflects a general form of temporal expectation as to the impeding stimulus (Praamstra et al., 2006; Cravo et al., 2011; Rohenkohl and Nobre, 2011) or whether they contain specific information relevant to the analysis of the incoming stimulus remains unclear. For instance, this uncertainty has led to the dichotomy of the “what” vs. “when” of prediction with regards to the informational content carried in an internal prediction (Arnal and Giraud, 2012; van Wassenhove, 2013).

In the current experimental design, a significant increase of auditory baseline activity about 100 ms following a color change was observed as a bilateral ramp up of activity until sound onset (DEV trials). The subsequent auditory evoked responses were significantly smaller in amplitude as compared to when sounds were not preceded by a visual transience (STD trials). This early phase-locked response—also observed in the alpha component—is consistent with prior reports in which desynchronized audiovisual events elicit a latency shifts in the evoked response and a decreased amplitude of the sensory evoked responses (van Wassenhove et al., 2005; Raij et al., 2010; Vroomen and Stekelenburg, 2010; van Wassenhove, 2013).

A strong decrease in alpha power has previously been reported to indicate temporal expectation (Praamstra et al., 2006; Rohenkohl and Nobre, 2011). Here, visual events were markedly predictive of “when” auditory onsets would occur irrespective of their location. Although the observed ramping activity preceding the sound onset was highly suggestive of stimulus predictability induced by the visual transience (DEV), the observed alpha increase appeared to be inconsistent with classic temporal expectation effects. The interplay between temporal prediction and expectation is thus unclear but one possibility is that bottom-up temporal predictions (ramping activity) may actively suppress auditory attention to the sound (alpha increase) in the context of the task-requirement. Accordingly, the alpha oscillations have been proposed to index pulsed-inhibitory processing (Händel et al., 2011; Jensen et al., 2012) in line with the selective enhancement of attended stimuli and inhibition of unattended stimuli in selective attention (Desimone and Duncan, 1995).

Several recent studies have reported an increase of alpha power in cortical regions encoding the non-attended space or sensory modality (Frey et al., 2014). Consistent with this recent study (Frey et al., 2014), a systematic alpha power increase was observed in both auditory cortices when participants were engaged in a visual counting task. This suggests that the increase alpha power observed in auditory cortices is an active suppression of incoming auditory information when engaged in a visual task. In a different study, Banerjee et al. (2011) reported that both auditory and visual spatial tasks induced lateralized increases in alpha power over parieto-occipital regions and these were ipsilateral to the attended side of the stimulus (or contralateral to the unattended side of the stimulus). Here, no such specific distinction was observed suggesting that attention to sounds was fully suppressed irrespective of their location and remained independent of eye position, when attention was allocated to visual inputs.

### Automaticity of the visual “when” modulation of auditory cortices

A change in visual color predicted the temporal onset of a sound with full certainty but with no certainty as to its specific location. The visual deviance did not elicit a typical mismatch response in the auditory evoked response which showed, to the contrary, a decrease in amplitude (albeit an increase preceding the occurrence of the sound). This pattern suggests that predictive mechanisms typically observed in oddball paradigms may be under attentional control.

In a recent study, increases in high gamma band responses were reported for unexpected auditory changes (unexpected vs. expected omissions) and were interpreted as indices of residual errors in a predictive coding scheme (Todorovic and de Lange, 2012). Here, systematic high gamma band increases were seen contralateral to the sound location irrespective of eye position: if gamma band response resulted from spatial prediction, hemispheric differences would have been predicted in the opposite directions. Additionally, significant effects of eye positions would have been observed. Hence the gamma signature observed here does not appear to result from a comparison process selective to spatial processing. Alternatively, this signature could be interpreted as a possible gain modulation of the auditory cortex responses as a function of the temporal prediction provided by the visual transience. Although the “when” prediction (Arnal and Giraud, 2012) or temporal expectations (Nobre et al., 2007) are often reported in low-frequency oscillatory activity (Praamstra et al., 2006; Rohenkohl and Nobre, 2011), recent hypotheses suggest an important role of the alpha/gamma coupling in the temporal organization of information processing (Lisman and Jensen, 2013). It should be noted that the gamma oscillatory component appeared to be mostly locked—not fully induced—suggesting a partial mediation by bottom-up visual inputs of the auditory gamma band response. This does not exclude possible attentional modulation in the gamma response (Siegel et al., 2012).

Altogether, our data failed to capture a consistent modulation of the auditory evoked responses as a function of eye positions in the absence of saccades, in the presence of visual transience and when attention was directed to visual events. However, systematic modulations of the auditory evoked and oscillatory responses were observed at the onset of the auditory stimuli when preceded by a visual transient change. This suggests the existence of a “when” prediction for the time of occurrence of the auditory stimulus irrespective of its location and thus, that temporal predictive information can automatically shape auditory cortical response and regulate gamma band activity. Hence, although attentional idling was observed in the unattended auditory cortices (as indexed by increased alpha power), temporal predictions were preserved.

### Conflict of interest statement

The authors declare that the research was conducted in the absence of any commercial or financial relationships that could be construed as a potential conflict of interest.
